# Accuracy of the persistent AKI risk index in predicting acute kidney injury in patients admitted to the intensive care unit for acute respiratory failure

**DOI:** 10.5935/2965-2774.20230141-en

**Published:** 2023

**Authors:** João Raphael Zanlorensi Glir, Rafaella Stradiotto Bernardelli, Amanda Christina Kozesinski-Nakatani, Rafael Alexandre de Oliveira Deucher, Mirella Cristine de Oliveira, Álvaro Réa-Neto

**Affiliations:** 1 Centro de Estudos e Pesquisa em Terapia Intensiva - Curitiba (PR), Brazil

**Keywords:** Acute kidney injury, Respiratory insufficiency, Renal replacement therapy, Prognosis, Death, Mortality, Intensive care units, COVID-19, Coronavirus infections, SARS-CoV-2

## Abstract

**Objective:**

To evaluate the accuracy of the persistent AKI risk index (PARI) in predicting acute kidney injury within 72 hours after admission to the intensive care unit, persistent acute kidney injury, renal replacement therapy, and death within 7 days in patients hospitalized due to acute respiratory failure.

**Methods:**

This study was done in a cohort of diagnoses of consecutive adult patients admitted to the intensive care unit of eight hospitals in Curitiba, Brazil, between March and September 2020 due to acute respiratory failure secondary to suspected COVID-19. The COVID-19 diagnosis was confirmed or refuted by RT-PCR for the detection of SARS-CoV-2. The ability of PARI to predict acute kidney injury at 72 hours, persistent acute kidney injury, renal replacement therapy, and death within 7 days was analyzed by ROC curves in comparison to delta creatinine, SOFA, and APACHE II.

**Results:**

Of the 1,001 patients in the cohort, 538 were included in the analysis. The mean age was 62 ± 17 years, 54.8% were men, and the median APACHE II score was 12. At admission, the median SOFA score was 3, and 83.3% had no renal dysfunction. After admission to the intensive care unit, 17.1% had acute kidney injury within 72 hours, and through 7 days, 19.5% had persistent acute kidney injury, 5% underwent renal replacement therapy, and 17.1% died. The PARI had an area under the ROC curve of 0.75 (0.696 - 0.807) for the prediction of acute kidney injury at 72 hours, 0.71 (0.613 - 0.807) for renal replacement therapy, and 0.64 (0.565 - 0.710) for death.

**Conclusion:**

The PARI has acceptable accuracy in predicting acute kidney injury within 72 hours and renal replacement therapy within 7 days of admission to the intensive care unit, but it is not significantly better than the other scores.

## INTRODUCTION

Acute kidney injury (AKI) has an incidence of 20 to 50% in the population hospitalized in intensive care units (ICUs), with an estimated mortality of 20%.^(1)^ In the intensive care setting, AKI may represent up to 60% of complications in patients with severe acute respiratory syndrome (SARS),^(2)^ making the early identification of these organ dysfunctions crucial for the clinical management of patients, whether to aid in decisions to prevent potential damage or to improve clinical procedures and/or to estimate prognoses.

In this scenario, despite the need to classify AKI, as widely established in the literature through Acute Kidney Injury-Kidney Disease: Improving Global Outcomes (AKI-KDIGO),^(3)^ the concept of renal angina becomes relevant because it prompts the early identification of patients at risk of developing renal injury - similar to cardiac angina, which precedes acute myocardial infarction - through predictive scores and/or biomarkers.^(4)^

Renal angina was initially addressed in the pediatric population in the second decade of this century as a way of predicting progression to AKI. Thus, scores such as the renal angina index (RAI) emerged in an attempt to quantify the probability of progression to AKI and the persistence of the disease.^(5)^ In the adult population, the concept of renal angina has been little explored so far.^(6-8)^ In an attempt to bring relevance to the concept of renal angina in the adult intensive care population, the persistent AKI risk index (PARI) was developed, which was validated in a Japanese database of critically ill patients. It reflects the small variations in serum creatinine, in addition to the clinical conditions at admission, such as the presence of hyperbilirubinemia, sepsis and ventilatory/hemodynamic support. The objective of the score is to predict the development and persistence of AKI (i.e., for more than 72 hours), the need for renal replacement therapy (RRT), and death.^(7)^

Although PARI is promising, it lacks validation for other diagnoses and clinical conditions at ICU admission, and its accuracy in the early identification of AKI is unknown. In this context, we conducted a diagnostic study to evaluate, in patients hospitalized for acute respiratory failure, the accuracy of PARI at predicting AKI at 72 hours after ICU admission as well as persistent AKI, RRT, and death until the 7th day of admission.

## METHODS

This cohort study was done on data from a prospective cohort of consecutive adult patients admitted with acute respiratory failure to the ICU of eight hospitals in Curitiba, Paraná, Brazil, between March 11 and September 13, 2020. Patients were covered by either the Unified Health System (SUS - *Sistema Único de Saúde*) or the Supplementary Health System.

The cohort study was approved by the Ethics Committee of the *Instituto de Neurologia de Curitiba* under protocol 3,000,353 on September 17, 2018, and the need for informed consent was waived due to the noninterventional study design and data collection (we only reviewed medical records without contacting the participants). All research procedures were conducted in accordance with the ethical standards of the local Ethics Committee and the 1975 Declaration of Helsinki, revised in 2000. The Standards for Reporting of Diagnostic Accuracy (STARD) guidelines were used to guide the writing of this study.

The cohort study included patients older than 18 years admitted to ICUs with acute respiratory failure secondary to suspected respiratory infection who had available results of a reverse transcription-real-time polymerase chain reaction (RT-PCR) test for the detection of severe acute respiratory syndrome coronavirus 2 (SARS-CoV-2) run on a nasopharyngeal swab. Patients were considered to have acute respiratory failure when they presented two or more of the following clinical and radiological criteria: (A) at least one flu-like illness, that is, cough, runny nose, fever, or sore throat; (B) at least two points on the modified quick Sepsis-related Organ Failure Assessment (qSOFA) (systolic blood pressure < 100mmHg, respiratory rate > 22bpm, lowered consciousness level with Glasgow coma scale score < 15 and/or pulse oxygen saturation < 93%); and (C) chest computed tomography suggestive of coronavirus disease 2019 (COVID-19) (ground-glass opacity and peripheral lesions distributed in both lungs) within the first 48 hours after admission.^(9)^

Data were systematically extracted from the electronic medical records of the patients, as well as from the medical records recorded daily on paper forms. Personal and clinical characteristics at ICU admission and daily clinical and laboratory data for the first 30 days in the ICU or until the outcome (discharge or death) in the ICU were collected from all records.

Excluded were patients who did not have creatinine, urine output, or a record of whether RRT was performed at least three mandatory times, which were at ICU admission, 24 hours, and 72 hours after admission; who were hospitalized in the ICU for less than 72 hours; who died less than 72 hours after admission to the ICU; who had creatinine greater than 4mg/dL; and who had previously known chronic kidney disease recorded in the medical records.

The sample was characterized by sex, age, confirmed diagnosis of COVID-19, presence of self-reported comorbidities, and the following data from the first 24 hours in the ICU: Acute Physiology and Chronic Health Evaluation (APACHE II) score, Sequential Organ Failure Assessment (SOFA) score, and the change in SOFA score (delta SOFA), use of vasoactive drugs (VAD), need for mechanical ventilation (MV), creatinine values and their change (delta creatinine), AKI-KDIGO stage, and presence of hyperbilirubinemia (bilirubin > 2mg/dL). The use of antibiotics in the first 48 hours, as well as nephrotoxic drugs (polymyxin B, colistin, gentamicin, amikacin, vancomycin, and/or antifungal drugs) within 2 and up to 6 days after ICU admission, is also described.

PARI was calculated from the following information: creatinine variation in the first 24 hours in the ICU (delta creatinine), total bilirubin, need for MV or VAD, and presence or absence of sepsis on admission.^(10)^ Sepsis was diagnosed in those patients who had SOFA increases ≥ 2 in 24 hours^(11)^ and were on antibiotics for ≥ 48 hours. To calculate PARI, each variable was assigned a weight: delta creatinine < 0.2mg/dL, score 1; ≥ 0.2mg/dL, score 2; ≥ 0.3mg/dL, score 4; ≥ 0.4mg/dL, score 10; presence of hyperbilirubinemia (Bt ≥ 2mg/dL) and sepsis, score 2 each; need for VAD or MV, score 4. If there was no aggravating condition, the score was assumed to be 1. PARI equaled delta creatinine multiplied by the sum of the other conditions, and the score could have the following values: 1, 2, 4, 6, 8, 10, 12, 16, 20, 24, 40, 60, and 80.^(7)^

AKI was defined according to the AKI-KDIGO, as follows: stage 1 if serum creatinine elevation was 1.5 - 1.9 times the baseline value or increased ≥ 0.3mg/dL in 48 hours or the urine output was < 0.5mL/kg/hour for 6 to 12 hours; stage 2 if serum creatinine was 2 - 2.9 times baseline or urine output was < 0.5mL/kg/hour for at least 12 hours; stage 3 if serum creatinine was ≥ 3 times baseline or ≥ 4mg/dL, or urine output was < 0.3mL/kg/h for at least 24 hours, or anuria lasted at least 12 hours, or RRT was started.^(3)^

The accuracy of PARI against that of delta creatinine alone and SOFA alone was evaluated as per Matsuura et al.,^(7)^ as well as against APACHE II, to predict the primary outcome of AKI (AKI-KDIGO 2 or 3) at 72 hours after admission to the ICU. We also calculated its accuracy at predicting the secondary outcomes: persistent AKI (AKI-KDIGO 2 or 3 for more than 72 hours), use of RRT, and death within 7 days after ICU admission.

### Statistical analysis

Categorical variables are presented as n (%), quantitative variables with normal distributions are presented as mean ± standard deviation, and quantitative variables without normal distributions are presented as mean, median, and interquartile range. Categorical variables were compared between groups with and without AKI (AKI-KDIGO 2 or 3) 72 hours after ICU admission using the chi-squared test or Fisher’s exact test, as appropriate. Quantitative comparisons between groups were performed by Student’s *t* test for independent samples when the data were normally distributed and by the nonparametric Mann‒Whitney test when the data were not normally distributed.

The accuracy of PARI, delta creatinine, SOFA, and APACHE II was evaluated using the receiver operating characteristic curve (ROC) method, whose results are described by area under the ROC curve and its confidence interval for each of the outcomes evaluated. The areas under the ROC curve of PARI, delta creatinine, SOFA, and APACHE II were compared by the DeLong method. The optimal PARI cutoff point for each outcome was that which maximized Youden’s statistic, and its sensitivity, specificity, and positive and negative predictive values are reported. Finally, the outcomes were compared between groups established by the optimal cutoff point identified in the study, as well as by the cutoff point of PARI ≥ 8 found by Matsuura et al.^(7)^

The same analyses described above were performed in the subgroups with and without COVID-19. The analyses were performed using IBM Statistical Package for Social Sciences (SPSS) software, version 28.0 (SPSS Inc., Chicago, Illinois, United States). The cutoff for statistical significance was 5%, and no values were imputed to correct missing data for any variable.

## RESULTS

**Figure 1:**
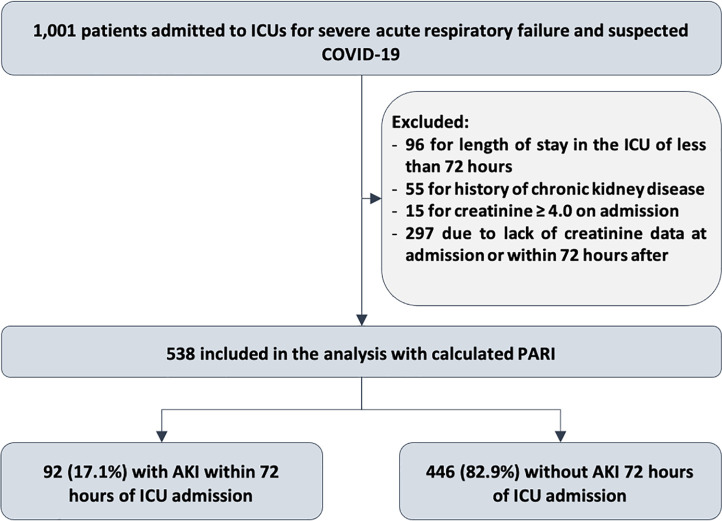
Flowchart of the sampling process.

All 1,001 patients in the cohort were considered for the study. Of these, 463 patients (54%) were excluded for meeting an exclusion criterion, with 538 patients sampled for the study, of which 82% had a confirmed diagnosis of COVID-19, and in 18%, this diagnosis was refuted (Figure 1).

ICU - intensive care unit; PARI - persistent AKI risk index; AKI - acute kidney injury.

The enrolled sample had a mean age of 62 ± 17 years, 54.8% were male, the median APACHE II score was 12, the median SOFA score at admission was 3, 83.3% had no renal dysfunction at admission, and fewer than 5% used nephrotoxic drugs in the first 7 days in the ICU. Table 1 shows these and other characteristics of the total sample, as well as the comparison between the groups with and without AKI (AKI-KDIGO 2 or 3) at 72 hours after admission (no score on AKI-KDIGO or AKI-KDIGO 1).

**Table 1:** Comparison of groups with the presence or absence of acute kidney injury (*Kidney Disease: Improving Global Outcomes* 2 or 3) at 72 hours

Variables	Total sample (n = 538)	No AKI in 72 hours (n = 446)	With AKI in 72 hours (n = 92)	p value
Male sex	295 (54.8)	248 (55.6)	47 (51.1)	0.490^*^
Age (years)	62 ± 17	61 ± 16	66 ± 16	0.010†
Comorbidities				
Heart disease	105 (19.5)	80 (17.9)	25 (27.2)	0.059^*^
SH	266 (49.4)	213 (47.8)	53 (57.6)	0.087^*^
Liver failure	9 (1.7)	9 (2)	0 (0)	0.369^*^
Cerebrovascular disease	25 (4.6)	21 (4.7)	4 (4.3)	1^*^
Diabetes	167 (31)	137 (30.7)	30 (32.6)	0.712^*^
HIV/AIDS	8 (1.5)	6 (1.3)	2 (2.2)	0.630^*^
Cancer	25 (4.6)	23 (5.2)	2 (2.2)	0.284^*^
Obesity‡	116 (38.5)	87 (36.1)	29 (48.3)	0.103^*^
Diagnosis of COVID-19	439 (81.6)	360 (80.7)	79 (85.9)	0.301^*^
APACHE II	14; 1 (8 - 18)	13; 12 (7 - 17)	19; 19 (12 - 25)	< 0.001§
PARI	6.2; 2 (1 - 6)	4.5; 2 (1 - 4)	14.9; 6 (2 - 20)	< 0.001§
PARI components				
Creatinine on admission	1.03; 0.88 (0.69 - 1.20)	0.97; 0.86 (0.68 - 1.15)	1.37; 1 (0.76 - 1.75)	< 0.001§
Delta creatinine	0.06; 0 (-0.1 - 0.17)	0.01; 0 (-0.1 - 0.13)	0.29; 0.16 (-0.01 - 0.46)	< 0.001§
Use of VAD at admission	98 (18.2)	73 (16.4)	25 (27.2)	0.018^*^
Use of MV on admission	145 (27)	101 (22.6)	44 (47.8)	< 0.001^*^
SOFA on admission	4; 3 (2 - 6)	4; 3 (2 - 5)	6; 6 (3 - 8)	< 0.001§
Antibiotic use during the first 48 hours	327 (60.8)	271 (60.8)	56 (60.9)	1^*^
Hyperbilirubinemia on admission	4 (0.7)	2 (0.4)	2 (2.2)	0.137^*^
AKI-KDIGO in the first 24 hours				< 0.001¶
Without AKI	448 (83.3)	388 (87)	60 (65.2)	
Stage 1	67 (12.5)	52 (11.7)	15 (16.3)	
Stage 2	19 (3.5)	6 (1.3)	13 (14.1)	
Stage 3	4 (0.7)	0 (0)	4 (4.3)	
Use of nephrotoxic drugs on the 2nd day in the ICU ||	9 (1.7)	6 (1.4)	3 (3.4)	0.174^*^
Use of nephrotoxic drugs on the 6th day of the ICU ||	24 (4.6)	16 (3.6)	8 (9.2)	0.042^*^
Length of stay in the ICU	11.6; 7 (5 - 14)	10.7; 7 (5 - 13)	16.0; 9.5 (6 - 20)	0.003§
Persistent AKI for up to 7 days	105 (19.5)	46 (10.3)	59 (64.1)	< 0.001^*^
RRT within 7 days#	27 (5)	10 (2.2)	17 (18.55)	< 0.001^*^
Mortality within 7 days	68 (12.6)	37 (8.3)	31 (33.7)	< 0.001^*^

AKI - acute kidney injury; SH - systemic arterial hypertension; APACHE II - Acute Physiology and Chronic Health Evaluation; PARI - persistent AKI risk index; VAD - vasoactive drug; MV - invasive mechanical ventilation; SOFA - Sequential Organ Failure Assessment Score; AKI-KDIGO - Acute Kidney Injury-Kidney Disease: Improving Global Outcomes; ICU - intensive care unit; RRT - renal replacement therapy. Obesity was defined as a body mass index ≥ 30; hyperbilirubinemia was defined as total bilirubin ≥ 2mg/dL. The following were considered nephrotoxic agents: polymyxin B, colistin, gentamicin, amikacin, vancomycin and/or antifungal agents.

* Fisher's exact testsignificance, p < 0.05; † Significance of Student 's t test for independent samples, p < 0.05; ‡ 227 missing data points in the total sample: 195 in the group without acute kidney injury within 72 hours, 32 in the group with acute kidney injury within 72 hours; § significance of the nonparametric Mann-Whitney test, p < 0.05; ¶ significance of the chi-squared test, p < 0.05; || 12 missing data points in the total sample: 7 in the group without acute kidney injury within 72 hours, 5 in the group with acute kidney injury within 72 hours; # 1 missing data point in the total sample and in the group without acute kidney injury at 72 hours. The results are expressed as n (%), mean ± standard deviation, or mean; median (interquartile range).

Patients with AKI within 72 hours of admission had significantly higher IAP values, admission creatinine, delta creatinine, APACHE II, and SOFA than those without AKI, as well as longer length of ICU stay, RRT use and mortality rate. (Table 1).

The groups were not different in sex, presence of comorbidities, diagnosis of COVID-19, hyperbilirubinemia at admission, or use of antibiotics or nephrotoxic drugs in the first 48 hours. Age was significantly higher in the AKI group, which group also had a higher proportion of patients needing MV and taking VAD, higher stages of AKI-KDIGO at admission, and more use of nephrotoxic drugs up to the 6th day in the ICU (Table 1).

**Figure 2:**
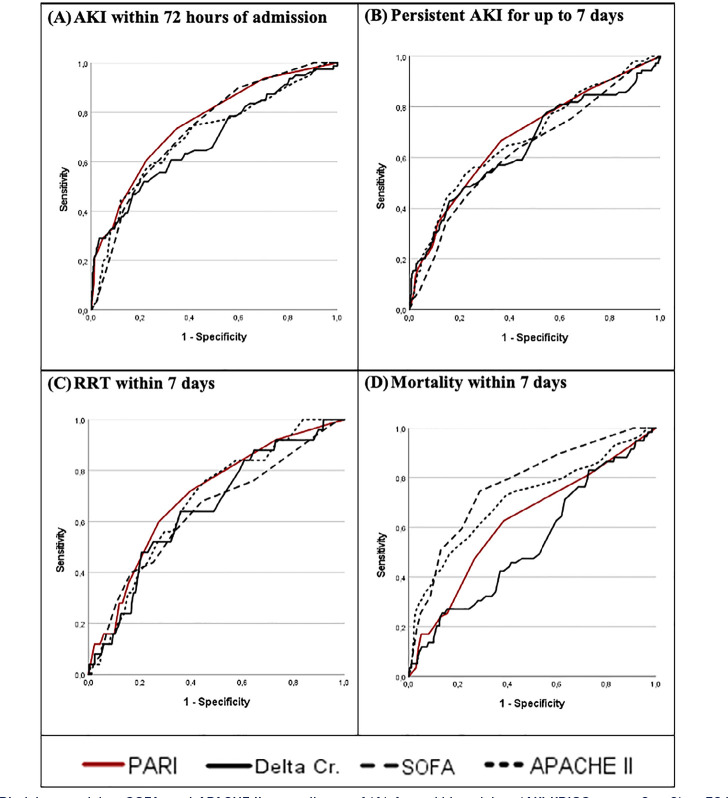
ROC curve of PARI, delta creatinine, SOFA, and APACHE II as predictors of (A) Acute kidney injury (AKI-KDIGO stages 2 or 3) ≤ 72 hours after admission; (B) acute kidney injury persisting for up to 7 days; (C) renal replacement therapy within 7 days; and (D) death up to the 7th day.

The accuracy values of PARI, delta creatinine, SOFA at admission, and APACHE II as predictors of AKI (AKI-KDIGO stage 2 or 3) 72 hours after admission, persistent AKI, need for RRT, and mortality up to the 7th day are presented in figure 2 and table 2, which also compare PARI with the other three methods.

**Table 2:** Comparison of the areas under the ROC curves of PARI, delta creatinine, SOFA, and APACHE II in the prediction of acute kidney injury (AKI-KDIGO stages 2 or 3) at 72 hours, persistent acute kidney injury, renal replacement therapy up to 7 days, and death up to 7 days

Total sample	Area under the ROC curve (95%CI)	p value *versus* PARI^*^
AKI in 72 hours (n = 538)		
PARI	0.751 (0.697 - 0.806)	-
Delta creatinine	0.674 (0.608 - 0.739)	0.013
SOFA	0.711 (0.655 - 0.767)	0.254
APACHE II	0.699 (0.637 - 0.761)	0.178
Persistent AKI for up to 7 days (n = 538)		
PARI	0.683 (0.624 - 0.742)	-
Delta creatinine	0.649 (0.585 - 0.713)	0.277
SOFA	0.631 (0.569 - 0.692)	0.136
APACHE II	0.681 (0.622 - 0.741)	0.960
RRT within 7 days (n = 537)		
PARI	0.710 (0.614 - 0.806)	-
Delta creatinine	0.671 (0.569 - 0.773)	0.458
SOFA	0.65 (0.536 - 0.764)	0.266
APACHE II	0.671 (0.577 - 0.766)	0.452
Mortality within 7 days (n = 538)		
PARI	0.638 (0.567 - 0.709)	-
Delta creatinine	0.552 (0.477 - 0.627)	0.033
SOFA	0.762 (0.700 - 0.824)	< 0.001
APACHE II	0.708 (0.636 - 0.781)	0.113

95%CI - 95% confidence interval; PARI - persistent AKI risk index; AKI - acute kidney injury; SOFA - Sequential Organ Failure Assessment Score; APACHE II - Acute Physiology and Chronic Health Evaluation; RRT - renal replacement therapy.

* Significance of the comparison of the area under the curve of the persistent AKI risk index with the other parameters for each of the three outcomes, using the DeLong method, p < 0.05.

AKI - acute kidney injury; RRT - renal replacement therapy; PARI - persistent AKI risk index; Cr - creatinine; SOFA - Sequential Organ Failure Assessment Score; APACHE II - Acute Physiology and Chronic Health Evaluation II.

PARI’s area under the ROC curve was higher than that of the three other methods. The evaluation of their predictive potential by the DeLong method showed that for AKI at 72 hours, PARI was better than delta creatinine but was not significantly different from SOFA or APACHE II. Regarding the analysis of persistent AKI and RRT within 7 days, there was no significant difference between the PARI and the other methods evaluated, even though the area under the ROC curve was also higher. Regarding death within 7 days, PARI was a better predictor than the delta creatinine value but less accurate than the SOFA score and similar to the APACHE II score (Figure 2 and Table 2).

PARI ≥ 4 was the best cutoff point to predict AKI (AKI-KDIGO stage 2 or 3) at 72 hours, persistent AKI, and death within 7 days, as identified by the Youden index, while a PARI ≥ 6 best predicted the use of RRT up to the 7th day. Table 3 lists the sensitivity, specificity, positive predictive value (PPV), negative predictive value (NPV), and accuracy of these two cutoff points and the cutoff point of PARI ≥ 8^(7)^ for the three outcomes investigated. PARI ≥ 4 had a sensitivity of more than 73% for identifying AKI (AKI-KDIGO 2 and 3) within 72 hours and RRT up to the 7th day. PARI ≥ 6 had a similar specificity for discriminating the four studied characteristics-with an accuracy > 70%-but, the particularly strong point was the high NPV at each cutoff point, greater than 86%.

**Table 3:** Sensitivity, specificity, positive predictive value, negative predictive value, and accuracy of the cutoff points of PARI ≥ 4, ≥ 6 and ≥ 8 for the outcomes studied

PARI ≥ 4	PARI (+) (n = 228)	PARI (-) (n = 310)	Sensitivity (95%CI)	Specificity (95%CI)	VPP (95%CI)	VPN (95%CI)	Accuracy (95%CI)
AKI up to 72 hours	68 (29.8)	24 (7.7)	73 (64.9 - 82.9)	64.1 (59.7 - 68.6)	29.8 (23.9 - 35.8)	92.3 (89.3 - 95.2)	65.8 (61.8 - 69.8)
Persistent AKI	70 (30.7)	35 (11.3)	66.7 (57.6 - 75.7)	63.4 (58.9 - 68.0)	30.7 (24.7 - 36.7)	88.7 (85.1 - 92.2)	64.1 (60.0 - 68.1)
RRT^*^	20 (8.8)	7 (2.3)	74.1 (57.5 - 90.6)	59.3 (55 - 63.6)	8.8 (5.1 - 12.4)	97.7 (96.1 - 99.4)	60.1 (55.9 - 64.2)
Mortality	44 (19.3)	24 (7.7)	64.7 (53.3 - 76.1)	60.9 (56.4 - 65.3)	19.3 (14.2 - 24.4)	92.3 (89.3 - 95.2)	61.3 (57.2 - 65.5)
PARI ≥ 6	PARI (+) (n = 159)	PARI (-) (n = 379)	Sensitivity (95%CI)	Specificity (95%CI)	VPP (95%CI)	VPN (95%CI)	Accuracy (95%CI)
AKI up to 72 hours	56 (35.2)	36 (9.5)	60.9 (50.9 -70.8)	76.9 (73 - 80.8)	35.2 (27.8 - 42.6)	90.5 (87.5 - 93.5)	74.2 (70.5 - 77.9)
Persistent AKI	54 (34.0)	51 (13.5)	51.4 (41.9 - 61.0)	75.8 (71.7 - 79.8)	34.0 (26.6 - 41.3)	86.5 (83.1 - 90.0)	71.0 (67.2 - 74.8)
RRT	17 (10.7)	10 (2.6)	63.0 (44.7 - 81.2)	72.2(68.3 - 76.1)	10.7 (5.9 - 15.5)	97.4 (95.7 - 99.0)	71.7 (67.9 - 75.6)
Mortality	33 (20.1)	35 (9.2)	48.5 (36.7 - 60.4)	73.2 (69.2 - 77.2)	20.8 (14.5 - 27.1)	90.8 (87.9 - 93.7)	70.1 (66.2 - 73.9)
PARI ≥ 8	PARI (+) (n = 85)	PARI (-) (n = 453)	Sensitivity (95%CI)	Specificity (95%CI)	VPP (95%CI)	VPN (95%CI)	Accuracy (95%CI)
AKI up to 72 hours	37 (43.5)	55 (12.1)	40.2 (30.2 - 50.2)	89.2 (86.4 - 92.1)	43.5 (33 - 54.1)	87.9 (84.9 - 90.9)	80.9 (77.5 - 84.2)
Persistent AKI	36 (42.4)	69 (15.2)	34.3 (25.2 - 43.4)	88.7 (85.7 - 91.7)	42.4 (31.8 - 52.9)	84.8 (81.5 - 88.1)	78.1 (74.6 - 81.6)
RRT^*^	10 (11.8)	17 (3.8)	37.0 (18.8 - 55.3)	85.3 (82.3 - 88.4)	11.8 (4.9 - 18.6)	96.2 (94.5 - 98)	82.9 (79.7 - 86.1)
Mortality	17 (20)	51 (11.3)	25.0 (14.7 - 35.3)	85.5 (82.4 - 88.7)	20 (11.5 - 28.5)	88.7 (85.8 - 91.7)	77.9 (74.4 - 81.4)

PARI - Persistent AKI Risk Index; 95% CI - 95% confidence interval; PPV - positive predictive value; NPV - negative predictive value; AKI - acute kidney injury; RRT - renal replacement therapy.

* 1 missing data in the PARI < 4 and PARI < 8 groups.

## DISCUSSION

The PARI performed better at predicting AKI at 72 hours after ICU admission than predicting AKI persisting for up to 7 days. This give us an earlier therapeutic window to be explored using PARI, with the aim of reinforcing the initial measures suggested by KDIGO.^(3)^

AKI is considered a complex disease with a significant impact on the mortality of hospitalized patients, especially those in the ICU, due to its high incidence of 20 - 40%.^(1,12,13)^ The mortality rate of patients who develop AKI in the ICU varies according to the severity of the injury (KDIGO 1, 2, or 3), the need for RRT, and the clinical profile of the patient, the probability of death increasing by up to six times when the patient has KDIGO 3 injury,^(14)^ by 2 - 3 times when there is simultaneous AKI with pulmonary dysfunction, and by 50% when the patient has AKI and sepsis, and organ dysfunction has the greatest impact on mortality in this population.^(15,16)^

Thus, a fundamental concept is that of renal angina,^(17)^ which, although not necessarily presenting clear clinical signs and symptoms, according to Goldstein et al.,^(4)^ can be defined as oliguria and/or changes in serum creatinine in a relevant clinical context in which there are risk factors such as age, diabetes, sepsis, cirrhosis, being in the postoperative period, and critical illness.

If renal angina persists, AKI develops, in which there is impairment of renal function - combined or not with structural damage. The AKI may be transient, lasting less than 72 hours, or persistent, lasting ≥ 72 hours, thus increasing the risk of developing acute kidney disease with all its complications and impacts on mortality, ICU length of stay, need for RRT, and evolution for chronic kidney disease.^(18,19)^

Therefore, it is essential to search for tools in the intensive care environment that will identify patients at risk of renal dysfunction and thus assist in their evaluation by subjecting them to a particular nephrotoxic drug and contrast tests and to exclude, with greater safety and quantitative accuracy, those with a lower risk of long-term kidney injury.^(13)^ There are already models that stratify the risk of adult patients for developing AKI by taking into account previous comorbidities and creatinine variations;^(20)^ however, their usefulness is still debated, and none is well established.

The development of scores to identify patients at risk of persistent renal angina began in the pediatric population and then spread to the adult population. Publications on the subject have increased in the last decade. These scores incorporate biomarkers such as kidney injury molecule 1 (KIM-1), liver-type fatty acid-binding protein (L-FABP), interleukins (ILs), and neutrophil gelatinase-associated lipocalin (NGAL). However, they still need further validation in heterogeneous populations.^(19-22)^

In this study, we used the PARI to analyze a specific profile of patients - those admitted to the ICU for acute respiratory failure. This is because there is a significant increase in mortality in the presence of both dysfunctions (renal and pulmonary)^(23)^ through a complex pathophysiological mechanism involving not only humoral and cellular responses but also cytokines (IL-8, IL-6, and tumor necrosis factor), which promote and perpetuate inflammation as well as hydrostatic and nonhydrostatic edema in the parenchyma lung.^(24)^ Thus, there is a characteristic vicious cycle in which lung injury impairs kidney function and vice versa. Another motivation for the analysis of this population was the fact that, in the baseline study establishing PARI,^(7)^ there was a small proportion of patients hospitalized for respiratory reasons - approximately 8% of the sample in both cohorts - in addition to the outbreak of the COVID-19 pandemic.

In our analysis, the PARI cutoff point of ≥ 4 was the one that best discriminated persistent AKI in patients admitted to the ICU for acute respiratory failure, lower than the ≥ 8 identified by Matsuura et al.^(7)^ The lower PARI score found in our study could mean that the population with acute respiratory failure is more likely to develop renal dysfunction in the first 7 days after ICU admission, reinforcing the lung-kidney crosstalk,^(23-25)^ and/or it could mean our population was not as severe at ICU admission, given their SOFA and APACHE II values.

Another fact that may have influenced the results found for the PARI cutoff point and the ROC analysis comparing it with the other predictors is that 80% of the study population had a diagnosis of COVID-19. There is no consensus about the relationship between COVID-19 and AKI. Some studies suggest that there COVID-19 causes no greater predisposition to renal dysfunction than other diseases of equivalent severity,^(26-28)^ and AKI might even evolve slower under the acute respiratory failure caused by COVID-19 than under other etiologies. In addition, ethnic, sociodemographic, and treatment factors^(25)^ (e.g., corticosteroids given for COVID-19 may reduce the risk of AKI) may have contributed to the difference in the PARI cutoff in this specific clinical context.

Another point to note is the NPV found. The use of PARI ≥ 4 in our sample yielded a NPV greater than 92%. Specifically, our patients with PARI < 4 had a 92.3% chance of not having AKI within 72 hours after ICU admission; an 88.7% chance of not developing persistent AKI; a 97.7% chance of not requiring RRT in the next 7 days; and a 92.3% chance of not dying in this period. Because PARI is a practical index to be implemented at the bedside, the acceptable sensitivity values reinforced by the optimal NPVs make it able to identify with apparent safety the individuals at lower risk of long-term renal injury.

This study has some limitations inherent to its design. The generalizability of the study results is restricted, as it covers a population of respiratory patients who were mostly diagnosed with COVID-19, and 46% of the population was excluded due to criteria similar to the baseline criteria used by Matsuura et al.^(7)^ or due to lack of data on renal function. In addition, it was necessary to consider the use of antibiotics for more than 48 hours after admission due to the period of the COVID-19 pandemic as an additional factor for distinguishing cases of infection in the diagnosis of sepsis. However, it is noteworthy that the sampling was consecutive and included several hospitals in the same city as well as public and private insurance policies. The cutoff point identified for PARI needs to be validated in other contexts in patients admitted to the ICU for acute respiratory failure.

This study identified a new possibility of outcome to be explored using PARI, in addition to suggesting that the cutoff point of the score may depend on the clinical context in which it is applied. Future studies should be done in different populations on the outcome of AKI ≤ 72 hours after admission.

## CONCLUSION

In a population of patients with severe acute respiratory failure, PARI showed acceptable accuracy for predicting the development of acute kidney injury within 72 hours and/or the need for renal replacement therapy up to the 7th day of hospitalization, but it had an unsatisfactory performance in predicting persistent acute kidney injury and death, for which it was no better than SOFA or APACHE II. This study found a lower PARI cutoff value than the one that validated PARI, suggesting that there are different cutoff points for specific populations and populations with different reasons for hospitalization in the intensive care setting.

## References

[1] Case J, Khan S, Khalid R, Khan A. (2013). Epidemiology of acute kidney injury in the intensive care
unit. Crit Care Res Pract.

[2] Darmon M, Clec’h C, Adrie C, Argaud L, Allaouchiche B, Azoulay E (2014). Acute respiratory distress syndrome and risk of AKI among
critically ill patients. Clin J Am Soc Nephrol.

[3] Kidney Disease: Improving Global Outcomes (KDIGO) Acute Kidney
Injury Work Group (2012). KDIGO clinical practice guideline for acute kidney
injury. Kidney Int Suppl.

[4] Goldstein SL, Chawla LS. (2010). Renal angina. Clin J Am Soc Nephrol.

[5] Basu RK, Zappitelli M, Brunner L, Wang Y, Wong HR, Chawla LS (2014). Derivation and validation of the renal angina index to improve
the prediction of acute kidney injury in critically ill
children. Kidney Int.

[6] Ortiz-Soriano V, Kabir S, Claure-Del Granado R, Stromberg A, Toto RD, Moe OW (2022). Assessment of a modified renal angina index for AKI prediction in
critically ill adults. Nephrol Dial Transplant.

[7] Matsuura R, Iwagami M, Moriya H, Ohtake T, Hamasaki Y, Nangaku M (2020). A simple scoring method for predicting the low risk of persistent
acute kidney injury in critically ill adult patients. Sci Rep.

[8] Basu RK, Kaddourah A, Goldstein SL, AWARE Study Investigators (2018). Assessment of a renal angina index for prediction of severe acute
kidney injury in critically ill children: a multicentre, multinational,
prospective observational study. Lancet Child Adolesc Health.

[9] Oliveira MC, Scharan KO, Thomés BI, Stradiotto Bernardelli R, Reese FB, Kozesinski-Nakatani AC (2023). Diagnostic accuracy of a set of clinical and radiological
criteria for screening of COVID-19 using RT-PCR as the reference
standard. BMC Pulm Med.

[10] Matsuura R, Srisawat N, Claure-Del Granado R, Doi K, Yoshida T, Nangaku M (2018). Use of the renal angina index in determining acute kidney
injury. Kidney Int Rep.

[11] Singer M, Deutschman CS, Seymour CW, Shankar-Hari M, Annane D, Bauer M (2016). The Third International Consensus Definitions for Sepsis and
Septic Shock (Sepsis-3). JAMA.

[12] Chawla LS, Bellomo R, Bihorac A, Goldstein SL, Siew ED, Bagshaw SM, Bittleman D, Cruz D, Endre Z, Fitzgerald RL, Forni L, Kane-Gill SL, Hoste E, Koyner J, Liu KD, Macedo E, Mehta R, Murray P, Nadim M, Ostermann M, Palevsky PM, Pannu N, Rosner M, Wald R, Zarbock A, Ronco C, Kellum JA, Acute Disease Quality Initiative Workgroup 16 (2017). Acute kidney disease and renal recovery: consensus report of the
Acute Disease Quality Initiative (ADQI) 16 Workgroup. Nat Rev Nephrol.

[13] Kellum JA, Sileanu FE, Bihorac A, Hoste EA, Chawla LS. (2017). Recovery after acute kidney injury. Am J Respir Crit Care Med.

[14] Hoste EA, Bagshaw SM, Bellomo R, Cely CM, Colman R, Cruz DN (2015). Epidemiology of acute kidney injury in critically ill patients:
the multinational AKI-EPI study. Intensive Care Med.

[15] Griffin BR, Liu KD, Teixeira JP. (2020). Critical care nephrology: core curriculum 2020. Am J Kidney Dis.

[16] Levy MM, Macias WL, Vincent JL, Russell JA, Silva E, Trzaskoma B (2005). Early changes in organ function predict eventual survival in
severe sepsis. Crit Care Med.

[17] Vanmassenhove J, Kielstein J, Jörres A, Biesen WV. (2017). Management of patients at risk of acute kidney
injury. Lancet.

[18] Perinel S, Vincent F, Lautrette A, Dellamonica J, Mariat C, Zeni F (2015). Transient and persistent acute kidney injury and the risk of
hospital mortality in critically ill patients: results of a multicenter
cohort study. Crit Care Med.

[19] Nagata K, Horino T, Hatakeyama Y, Matsumoto T, Terada Y, Okuhara Y. (2021). Effects of transient acute kidney injury, persistent acute kidney
injury and acute kidney disease on the long-term renal prognosis after an
initial acute kidney injury event. Nephrology (Carlton).

[20] Cruz DN, Ferrer-Nadal A, Piccinni P, Goldstein SL, Chawla LS, Alessandri E, Belluomo Anello C, Bohannon W, Bove T, Brienza N, Carlini M, Forfori F, Garzotto F, Gramaticopolo S, Iannuzzi M, Montini L, Pelaia P, Ronco C, NEFROINT Investigators (2014). Utilization of small changes in serum creatinine with clinical
risk factors to assess the risk of AKI in critically ill
adults. Clin J Am Soc Nephrol.

[21] Chawla LS, Goldstein SL, Kellum JA, Ronco C. (2015). Renal angina: concept and development of pretest probability
assessment in acute kidney injury. Crit Care.

[22] Stanski NL, Wong HR, Basu RK, Cvijanovich NZ, Fitzgerald JC, Weiss SL (2021). Recalibration of the renal angina index for pediatric septic
shock. Kidney Int Rep.

[23] McNicholas BA, Rezoagli E, Pham T, Madotto F, Guiard E, Fanelli V, Bellani G, Griffin MD, Ranieri M, Laffey JG, ESICM Trials Group and the Large observational study to UNderstand
the Global impact of Severe Acute respiratory FailurE (LUNG SAFE)
Investigators (2019). Impact of early acute kidney injury on management and outcome in
patients with acute respiratory distress syndrome. Crit Care Med.

[24] Teixeira JP, Ambruso S, Griffin BR, Faubel S. (2019). Pulmonary consequences of acute kidney injury. Semin Nephrol.

[25] McNicholas BA, Rezoagli E, Simpkin AJ, Khanna S, Suen JY, Yeung P, Brodie D, Li Bassi G, Pham T, Bellani G, Fraser JF, Laffey J, CCCC Consortium (2023). Epidemiology and outcomes of early-onset AKI in COVID-19-related
ARDS in comparison with non-COVID-19-related ARDS: insights from two
prospective global cohort studies. Crit Care.

[26] Cheng Y, Luo R, Wang K, Zhang M, Wang Z, Dong L (2020). Kidney disease is associated with in-hospital death of patients
with COVID-19. Kidney Int.

[27] Park BD, Faubel S. (2021). Acute kidney injury and acute respiratory distress
syndrome. Crit Care Clin.

[28] Schaubroeck H, Vandenberghe W, Boer W, Boonen E, Dewulf B, Bourgeois C (2022). Acute kidney injury in critical COVID-19: a multicenter cohort
analysis in seven large hospitals in Belgium. Crit Care.

